# Real-Time Monitoring the Cytotoxic Effect of Andrographolide on Human Oral Epidermoid Carcinoma Cells

**DOI:** 10.3390/bios12050304

**Published:** 2022-05-06

**Authors:** Heng-Yi Liao, Chun-Chung Huang, Shih-Chi Chao, Chien-Ping Chiang, Bo-Hsuan Tang, Shiao-Pieng Lee, Jehng-Kang Wang

**Affiliations:** 1 Graduate Institute of Life Sciences, National Defense Medical Center, Taipei 114201, Taiwan; ian25520814@gapps.ndmctsgh.edu.tw; 2 Department of Chemistry, Tamkang University, New Taipei City 112304, Taiwan; 160662@mail.tku.edu.tw; 3 Institute of Oral Sciences, Chung Shan Medical University, Taichung 40201, Taiwan; h5l4g4fu6123@gmail.com; 4 Department of Medical Research and Education, Lo-Hsu Medical Foundation, Lotung Poh-Ai Hospital, Yilan 265501, Taiwan; 5 Department of Biochemistry, National Defense Medical Center, Taipei 114201, Taiwan; chiang@mail.ndmctsgh.edu.tw; 6 Department of Dermatology, Tri-Service General Hospital, Taipei 114202, Taiwan; 7 Information Technology and Management, Shih-Chien University, Taipei 104336, Taiwan; a106280113@gm1.usc.edu.tw; 8 Division of Oral and Maxillofacial Surgery, Department of Dentistry, Tri-Service General Hospital, Taipei 114202, Taiwan

**Keywords:** oral squamous carcinoma, andrographolide, electric cell-substrate impedance sensing

## Abstract

Andrographolide is an active diterpenoid compound extracted from *Andrographis paniculata*. It exhibits antiinflammatory and anticancer effects. Previous studies show that it is non-toxic to experimental animals. The leading causes of cancer are chronic inflammation and high blood glucose. This study determines the cytotoxic effect of andrographolide on cellular morphology, viability, and migration for human oral epidermoid carcinoma cell Meng-1 (OEC-M1). We use electric cell-substrate impedance sensing (ECIS) to measure the subsequent overall impedance changes of the cell monolayer in response to different concentrations of andrographolide for 24 h (10–100 µM). The results for exposure of OEC-M1 cells to andrographolide (10–100 µM) for 24 h show a concentration-dependent decrease in the overall measured resistance at 4 kHz. AlamarBlue cell viability assay and annexin V also show the apoptotic effect of andrographolide on OEC-M1 cells. A reduction in wound-healing recovery rate is observed for cells treated with 30 μM andrographolide. This study demonstrates that ECIS can be used for the in vitro screening of anticancer drugs. ECIS detects the cytotoxic effect of drugs earlier than traditional biochemical assays, and it is more sensitive and shows more detail.

## 1. Introduction

The oral cancer incidence rate in Taiwan is much higher than the global incidence rate. Bad habits such as chewing betel nut, smoking, and drinking alcohol increase the risk of developing oral cancer [1]. In 1995, epidemiological studies by Professor Ge Yingqin at Kaohsiung Medical University showed that individuals who use tobacco, alcohol, and betel nut have a 123-times greater risk of developing oral cancer than those who do not [2]. Too much-refined carbohydrate intake can cause chronic inflammation [3] and abnormal proliferation of malignant cells, which produces cancer invasion or metastasis to the distal region. Oral cancer can develop from the oral and oropharyngeal structures. The histology of cancer cell types includes squamous carcinoma, verrucous carcinoma, salivary gland cancer, and malignant melanoma. The most common cell type is squamous cell carcinoma [4].

The most undesirable result of cancer is metastasis. Oral cancer metastasizes in the lymphatic system around the neck, such as the jaw, chin, upper and middle neck. It can also metastasize to the lung, liver, bone, and lymphoid tissue under the clavicle. Surgical resection treats oral cancer, combined with cervical lymph node dissection surgery, followed by postoperative chemotherapy or radiotherapy [5]. Patients must then have intensive and regular follow-up examinations. Advanced oral cancer has a poor prognosis, which creates a significant medical and societal burden, so alternative cancer therapies are required. Chemotherapy is administered orally, by intravascular injection, or by intraperitoneal infusion. It is a systemic treatment transported by the circulatory system to the entire body. It is commonly used to remove residual cancer cells after surgery to control the growth or alleviate the disease’s symptoms and prevent metastasis. Chemotherapy interferes with the synthesis of DNA, RNA, or proteins to disrupt cell cycle progression, inhibits cancer cells’ growth, and increases the effectiveness of surgery and radiation therapy.

Apoptosis is a natural phenomenon, which is also called programmed death [6]. It is different from necrosis in that apoptosis does not induce inflammation. Apoptosis is a type of cell suicide [7]. Many pathways for apoptosis have been identified. Many studies seek the causes of apoptosis in cancer cells by using biochemical assays to identify cancer cells and normal cells and to control the growth and death of cancer cells [8].

The Bcl-2 family of apoptosis proteins is a key regulator of apoptosis, and family members also promote and inhibit apoptosis [9,10,11,12,13]. The apoptosis of Bax and Bak promotes ANT and VDAC interaction, and most studies focus on the function of the Bcl-2 family in the mitochondria and cytoplasm. Studies of Bcl-2 family members show a correlation between the family members that promote and inhibit apoptosis. The process of apoptosis uses the mitochondrial pathway. Studies of the caspase family began with studying the effect of cell death on C. elegans [14]. Apopain is the cell death enzyme [15], and PARP is the final product of death [16]. The synthesis of caspases in cells is non-active in the zymogen state, but activation allows it to function. When oral cancer occurs, p53, known as the guardian of the genome, triggers a series of apoptotic reactions [17]. If the DNA is damaged, the *TP53* gene inhibits the cell cycle from G1 to the S phase [18]. If cells lack p53, Bcl-2 expression increases, and BAX expression decreases, so BAX expression increases when cancer cells are apoptotic [19]. The downstream products, Caspase 3 and PARP, are cleaved by apoptosis and are indicators of apoptotic oral cancer cells [20], but the mechanism for the interaction between apoptotic is currently unclear.

Natural compounds are an effective anticancer treatment, and andrographolide is effective. Andrographolide is extracted from the annual herb plant *Andrographis paniculata*. *Andrographis paniculata* is known as the king of bitterness [21]. The formula for the diterpenoid lactone compounds is C_20_H_30_O_5_. Andrographolide is a diterpene lactone with a molecular weight of 350.46 kDa [21] and is one of the main active ingredients of *Andrographis paniculata* [22]. Andrographolide has been reported to have an anticancer effect by inhibiting translocation of DNA [23] and exhibits high antiinflammatory activity [24], anti-malarial activity, and anti-allergy activity. It also protects against liver injury in animal models, is anti-viral [24] and hyperglycemic [25], and regulates the immune response [26]. Andrographolide reduces ROS (reactive oxygen superoxide) production in human neutrophils [27] and decreases the inflammation of the ovalbumin-induced allergic reaction in lung disease [28]. Asian societies have used andrographolide for centuries as a Traditional Chinese Medicine to treat inflammation and infection [29]. Recent studies show that it can be used to treat diabetes mellitus [23], has an anticancer capacity [26,30], and exhibits low toxicity [31]; thus, it is suited for use in cancer therapy.

In biomedical research, electric cell-substrate impedance sensing (ECIS) has been used to study cell behaviors in cell culture. ECIS measures the complex impedance spectrum of adherent cells that grow on gold arrays. ECIS can sensitively monitor morphological changes of adherent cells in response to a variety of stimuli under physiological and pathological conditions [32]. The most common application of ECIS is to monitor the attachment and spreading of cells and the barrier function of a cell monolayer by measuring changes over time in impedance [33]. In this study, changes in morphology and migration of OEC-M1 cells in response to different concentrations of andrographolide were measured by ECIS. Two standard cytotoxicity methods, alamarBlue viability and annexin V/7-AAD binding assays, were performed in parallel to assess the cellular responses of the OEC-M1 cells. A comparison of the experimental data for an OEC-M1 covered electrode with the calculated values for a suitable cell-electrode model was used to calculate morphological parameters, such as junctional resistance between adjacent cells (Rb) and the average separation between the basolateral cell surface and the substratum (h) and the capacitance of the cell membrane (Cm) [34]. This technique allows a more sensitive measurement of apoptosis-induced morphological changes.

## 2. Materials and Methods

### 2.1. Cell Culture and Reagent Preparation

The OEC-M1 cell line was a kind gift from Professor Ching-Liang Meng at National Defense Medical Center. Cells were maintained in RPMI 1640 medium (Thermo Fisher Scientific, Waltham, MA, USA) containing 10% fetal bovine serum, 100 U/mL penicillin, and 100 μg/mL streptomycin at 37 °C/5% CO_2_. The cells were routinely sub-cultured when a confluence reached about 80–90%. At the 24th h after seeding, different concentrations of andrographolide were added until the 48th h. Andrographolide was purchased from Millipore Sigma (Billerica, MA, USA) and dissolved in dimethyl sulfoxide (DMSO) (Millipore Sigma, Billerica, MA, USA).

### 2.2. Impedance Measurement Using ECIS

ECIS Zθ instrument, electrode arrays, and acquired software for ECIS measurement were obtained from Applied Biophysics (Troy, NY, USA). The basic setup of ECIS is shown schematically in Figure S1. We cultured OEC-M1 cells on the electrode wells of the 8W1E array at a density of 1.25 × 10^5^ cells/cm^2^. Each array consists of eight wells that have a bottom area of 0.8 cm^2^ and contain one small gold film electrode with a 250 μm diameter and one much larger counter electrode. Confluency was reached 24 h after seeding, and cells were treated with each of the four different anticancer drugs for an additional 24 h. To measure the changes in cell morphology, the impedance of each cell-covered electrode was measured at 25 different frequencies ranging from 31.25 Hz to 100 kHz. The impedance was measured for a cell-free electrode and the same electrode covered with OEC-M1 cells. The exact frequency-scan measurements were made before and after cells were exposed to different concentrations of anticancer compounds.

A comparison of the experimental data for a cell-covered electrode with the calculated values using a suitable cell-electrode model is used to determine morphological parameters such as the junctional resistance between adjacent cells (Rb) and the average separation between the basolateral cell surface and the substratum (h), and the capacitance of the cell membrane (Cm) can be obtained. The cell–electrode model to calculate Rb, h, and Cm was described in a previous study [34]. To detect cell micromotion, impedance time series data of each electrode well were taken every second at 4 kHz until 2048 points were acquired. To compare the different levels of impedance fluctuations obtained under the influence of different compounds and concentrations, the variance for the 32-point sections (Var32) was used. Each data point in the 2048-point data set was first divided by its average value in this numerical analysis. Var32 was obtained by taking the average of the 64 variance values calculated from each 32-point section of the normalized 2048 data points [35].

### 2.3. AlamarBlue Viability Test

AlamarBlue cell viability reagent is used to assess cell viability. The viability reagent used the reducing power of living cells to measure the proliferation of various human cell lines. It is used to establish the relative cytotoxicity of agents within different chemical classes.

OEC-M1 cells were both exposed to andrographolide, and the viability reagent was added to two different 96-well plates with OEC-M1 cells and incubated for 4 h at 37 °C. The fluorescence signals for the 96-wells plate were then measured. All data were processed using the following equation.

Reduction of alamarBlue reagent = [(Experimental RFU value − Negative control RFU value)/(100% reduced positive control RFU value − Negative control RFU value)] × 100.

### 2.4. Annexin V/7-AAD Binding Assay

Annexin V staining was used to identify and measure the number of apoptotic cells. OEC-M1 cells (2 × 10^5^ to 5 × 10^5^ cells/mL) were treated according to the manufacturer’s instructions for the annexin V kit. The ability of different treatments to induce apoptosis against OEC-M1 cells was assessed by flow cytometry using a Muse^®^ Annexin V and Dead Cell Assay Kit (Millipore, Billerica, MA, USA).

To determine the ability of the different treatments to induce apoptosis against OEC-M1 cells, cells were plated in 24-well plates at a density of 5 × 10^4^ cells per well. After 24 h of incubation, cells were incubated with initial concentrations of andrographolide of 10, 30, 55, and 100 µM for 24 h. After 24 h, the cells were washed with PBS, trypsinized, and centrifuged at 1200 rpm for 10 min. After centrifugation, 1 × 10^5^ cells were stained according to the manufacturer’s instructions and analyzed using a Guava EasyCyte Plus Flow Cytometer (Millipore, Billerica, MA, USA). The degree of apoptosis is quantified in terms of the percentage of annexin V-positive cells.

### 2.5. Statistical Analysis

Statistical analysis uses a Student’s *t*-test and one-way ANOVA. All data are expressed as means ± SD and means ± SEM. The level of significance is *p* < 0.05.

## 3. Results

### 3.1. The Effects of Andrographolide on Cell Viability and Morphology in OEC-M1 Cells

**Figure 1:**
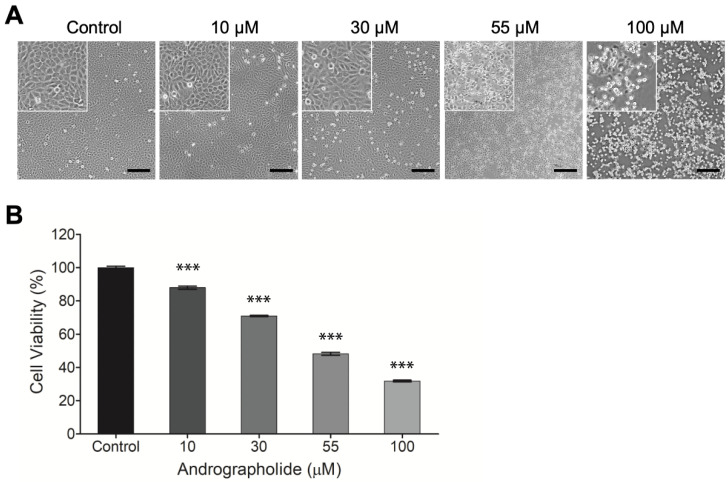
(**A**) Typical phase-contrast images of OEC-M1 cells cultured in 24-well cell culture plates 24 h after exposure to different concentrations (0, 10, 30, 55, and 100 μM) of andrographolide. Cell number decreases as compound concentration increases. Cells exhibit a perceptibly distressed and shrunken morphology 24 h after exposure to 100 μM of andrographolide. Images inside the top left small boxes are shown in 5x magnification. Scale bar = 200 µm. (**B**) Change in cell viability of OEC-M1 cells after treatment with andrographolide for 24 h at the indicated concentrations. Cell viability was evaluated by the alamarBlue assay. All treatments at higher concentrations significantly reduced cell viability relative to the control groups. Five independent experiments were performed (*n* = 5), and the results are shown as means ± SEM. *** *p* < 0.001.

OEC-M1 cells were incubated for 24 h, and then different concentrations of andrographolide (0, 10, 30, 55, and 100 μM) were added for 24 h. After 24 h, the results show that the highest concentration of andrographolide (100 μM) is cytotoxic for OEC-M1 cells. The IC50 value for OEC-M1 is 55 µM. Andrographolide has a concentration-dependent effect on OEC-M1 cells. The morphological image for the highest concentration of andrographolide (100 μM) shows that all cell membranes are damaged (Figure 1a).

The cytotoxicity of andrographolide on OEC-M1 cells was assessed using the alamarBlue assay. Figure 1b shows that the negative control groups have no toxic effect on the cells: The same values for all experiments were observed. There is a decrease in cell viability with the increase in drug concentrations from 10 to 100 µM. This result shows that OEC-M1 cells are sensitive to the treatment of andrographolide.

### 3.2. Real-Time Impedance Monitoring of OEC-M1 Cells Attachment and Spreading

**Figure 2:**
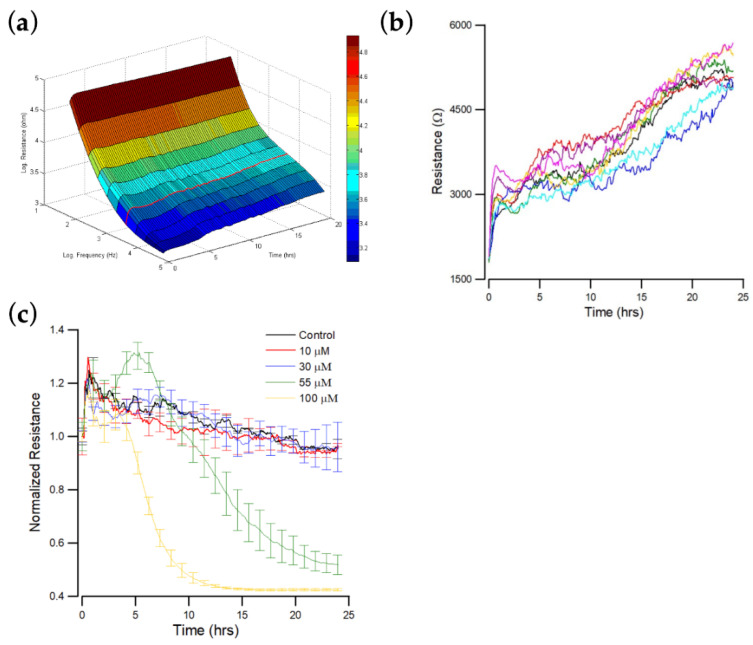
ECIS measurement of OEC-M1 cell attachment and spreading. At time zero, OEC-M1 were inoculated in an ECIS, giving a final cell density of 1.25 × 10^5^ cells per cm^2^. (**a**) Three-dimension representation of the electrical resistance as a function of frequency and time during the attachment and spreading of OEC-M1 cells on the sensing electrode. The red curve denotes the time-dependent resistance measured at 4 kHz. (**b**) Changes in resistance as a function of time measured at 4 kHz. Data were collected from eight independent electrodes for 24 h. These data are highly reproducible and similar to the red curve shown in (**a**). (**c**) Cells were inoculated into electrode-containing wells and allowed to develop into confluent layers for approximately 24 h. Andrographolide that was diluted in DMSO was then added to final concentrations of 10 μM (red), 30 μM (blue), 55 μM (green), 100 μM (yellow), and control (black), and the resultant changes in resistance were measured. Data were collected for 24 h after the addition of andrographolide. Only the resistance data at 4 kHz are shown.

The resistance spectra for cell-free and cell-covered electrodes are compared to determine the ultimate response frequency for a specific cell type. The impedance of OEC-M1 cells is measured at 25 different frequencies, from 31.25 Hz to 100 kHz, after cell attachment. The optimal frequency for OEC-M1 cells is 4 kHz (Figure 2a).

Figure 2b shows the resistance data measured at 4 kHz as a function of time, where 4 kHz is the optimal detection frequency for assessing OEC-M1. When cells attach and spread on the sensing electrode, their membranes interfere with the space directly above the electrode that is available for current flow. OEC-M1 grows as a confluent monolayer, so the cell-covered electrode’s measured resistance is relatively high at approximately 5–6 kΩ at 4 kHz. When the cells fully spread, the measured impedance fluctuates because the cells are in constant motion, so current flow changes. The final average impedance values and the fluctuations are characteristic of this cell type.

### 3.3. Effect of Andrographolide on the Time Course of Overall Resistance

The effect of different concentrations (0, 10, 30, 55, and 100 μM) of andrographolide on the total resistance of OEC-M1 monolayers over 24 h was measured. Impedance measurements were also performed at 11 different frequencies (62.5 Hz–64 kHz). Sixteen consecutive electrodes were used with 11 data points for each well requiring 10 s. The impedance of each electrode well was measured every 160 s, but fluctuations were observed on each curve at different levels. Figure 2c shows typical resistance data measured at 4 kHz for each andrographolide concentration. A rapid resistance decrease was observed at the highest concentration: 100 μM. This drop reached ~40% of the initial resistance value about 10 h after addition. The resistance decrease for the 55 μM treatment is slower but still significant, reaching ~50% of the initial resistance value about 25 h after addition. No significant differences were observed among the three lowest concentrations: 0, 10, and 30 μM.

### 3.4. Effect of Andrographolide on the Morphological Parameters of OEC-M1 Cells

**Figure 3:**
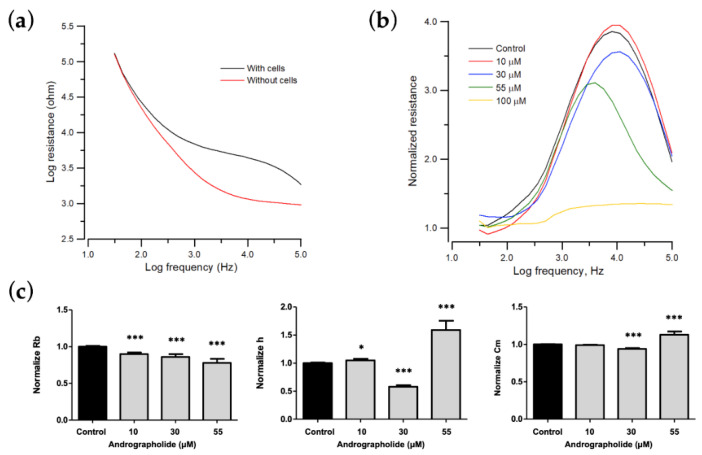
Change in morphological parameters of confluent OEC-M1 cells layers after challenge with different concentrations of andrographolide. (**a**) Resistance data as a function of frequency for cell-free (red line) and cell-covered (black line) electrodes. (**b**) Normalized resistance curves as a function of frequency using a frequency-scan measurement 24 h after the addition of andrographolide to confluent OEC-M1 cell layers at concentrations of 10 μM (red), 30 μM (blue), 55 μM (green), 100 μM (yellow), and control (black). (**c**) Changes in normalized junction resistance between cells, average cell–substrate separation, and membrane capacitance of confluent OEC-M1 cell layers after challenge with different concentrations of andrographolide. Six independent frequency scan experiments were performed (*n* = 6). Two or three wells were measured for each drug concentration for each experiment. Values are shown as means ± SEM. * *p* < 0.05, *** *p* < 0.001. Each value is expressed as a percentage of the control.

When cells attach and spread on the sensing electrodes, the main current does not pass through the insulating cell membrane, so it must flow around the cells. Reducing the area available for the current flow increases the system’s impedance. Minor cell–electrode interaction changes due to cell motion cause the impedance to fluctuate with time. Figure 3a shows the measured resistance as a function of frequency for a cell-free electrode and the same electrode cover with OEC-M1 cells. Different cell types have the maximum responses at different frequencies. Therefore, when monitoring cellular responses to toxins, the AC signal is set at the specific frequency that produces the most significant response to a change in impedance due to cell motion and metabolic activity. As the control curve shows in Figure 3b, the normalized resistance spectra display as biphasic curves, with a peak value at 4 kHz.

To determine the cytotoxic effect on morphological changes of OEC-M1 cells, especially on cell–cell and cell–substrate contacts, frequency-scan measurements were performed on cell-covered electrodes 24 h after exposure to different concentrations of andrographolide. Normalized resistance is calculated as a function of frequency by dividing the cell-covered resistance that is measured at various frequencies with the corresponding cell-free value (Figure 3b). For the normalized resistance spectrum for the control cells (black lines in Figure 3b), the ratio is about 1.1 at 31.5 Hz and increases as the frequency increases to a peak value of approximately 3.7 at 4 kHz. It then decreases as frequency increases to a value of 2 at 100 kHz. This biphasic feature of the normalized resistance spectrum shows that impedance measurements at 4 kHz sensitively reflects morphological changes in OEC-M1 cells. After the frequency-scan data of the control group were fitted with the cell–electrode model calculation, the junctional resistance between cells (Rb), the average cell–substrate separation (h), and the membrane capacitance (Cm) of the control OEC-M1 cells were 0.96 ± 0.05 Ω⋅cm^2^, 74 ± 3 nm, and 2.6 ± 0.1 μF/cm^2^ (*n* = 22), respectively.

The morphological parameters in response to each drug concentration are shown in Figure 3 and are expressed as a percentage of the control to allow comparison. Many OEC-M1 cells detach after 24 h of exposure to a concentration greater than 100 μM, so the model cannot fit the frequency-scan data for concentrations of more than 100 μM. For a concentration of andrographolide of 0, 10, 30, and 55 μM, the value for Rb decreases, and Cm increases in a concentration-dependent manner. There are unique phenomena at a concentrations of 30 μM and 55 μM. The value of h decreases at 30 μM but increases at 55 μM (Figure 3c).

### 3.5. A Wound Healing Migration Assay Using ECIS

In order to determine the concentration at which andrographolide inhibits the migration of OEC-M1 cells, the cells were firstly inoculated into electrode-containing wells for approximately 24 h. Confluent cells were then treated with different concentrations of andrographolide for another 24 h, and an ECIS wound healing assay was then performed.

**Figure 4:**
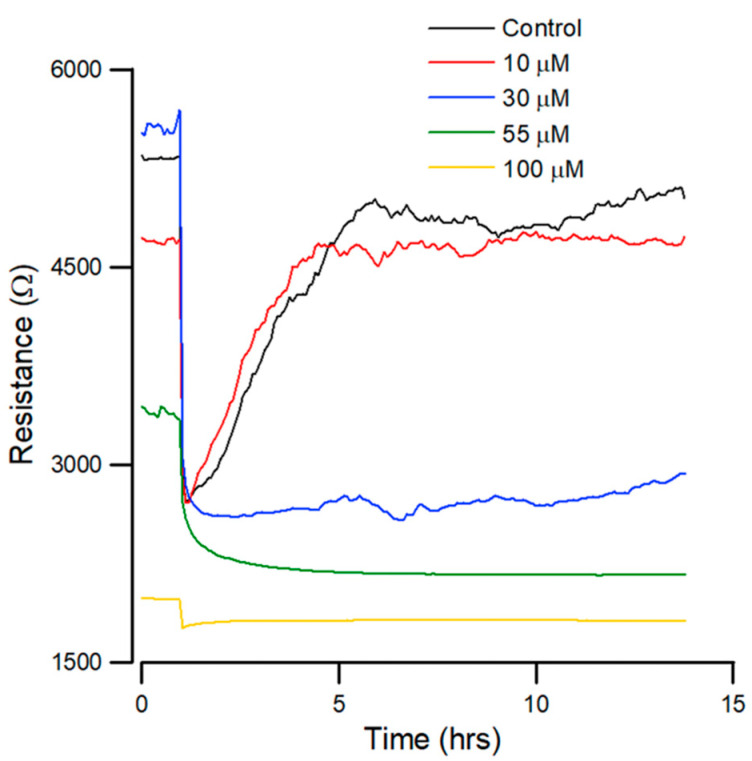
Effect of andrographolide on the wound healing migration of OEC-M1 cells, as measured by ECIS. Wound healing assay of andrographolide at concentrations of control (black), 10 (red), 30 (blue), 55 (green), and 100 µM (yellow) (*n* = 5).

The resistance was measured at 4 kHz for 14 h after wounding. Figure 4 shows that the cells are electrically wounded at t = 0.5 h, and data acquisition was briefly suspended for 6 min. The resistance values decrease to about 1.5–3 kΩ post-wounding, indicating cell death and detachment from microelectrodes. Data acquisition was then restarted at t = 0.6 h.

Figure 4 shows that the recovery curves for andrographolide have a different profile than that for the control. At lesser concentrations, there is no noticeable inhibition of migration. At higher concentrations of 55 µM, wound healing migration is completely inhibited after exposure to the drug for 24 h. In addition, 30 µM of andrographolide has an inhibitory effect on wound healing migration.

### 3.6. Effect of Andrographolide on OEC-M1 Micromotion

Previous studies show that cell movements in confluent layers detected via ECIS correlate with cell metabolic activity [34,36]. To detect subtle changes in the time-series resistances against different concentrations of andrographolide, rapid time collection (RTC) measurements were performed at 4 kHz after 24 h exposure of OEC-M1 to andrographolide. To quantitate cellular micromotion, impedance data for each well were measured every second until 2048 points were obtained, and then the next well was measured [35,36].

**Figure 5:**
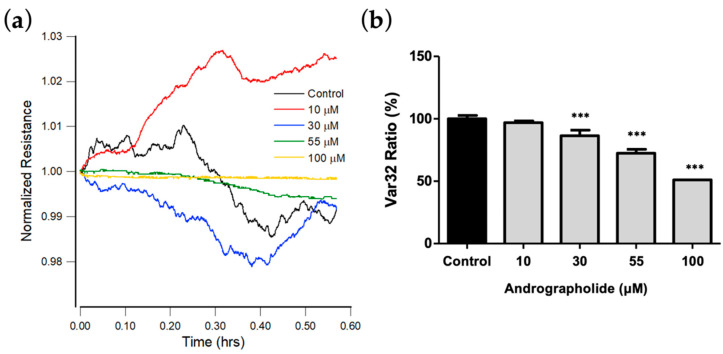
(**a**) Normalized resistance data recorded 24 h after the addition of medium containing andrographolide or medium alone to a confluent OEC-M1 layer to a final concentration of 10 μM (red), 30 μM (blue), 55 μM (green), 100 μM (yellow), and control (black). Each curve has data points at 1 s intervals. (**b**) Var32 analysis of OEC-M1 micromotion after exposure to andrographolide for 24 h at the indicated concentrations. Values are shown as means ± SEM. *** *p* < 0.001. Each value is expressed as a percentage of the control.

Figure 5a shows the plot for resistance data normalized to the value at the start of each run. The results show that the micro-motion of OEC-M1 is significantly inhibited at a concentration of andrographolide of more than 55 μM. Figure 5b shows the Var32 values for these time-series data. There is a significant dose-dependent decrease for an andrographolide concentration of 30 μM and higher.

### 3.7. Apoptotic Profile of OEC-M1 Cells after Drug Treatment

**Figure 6:**
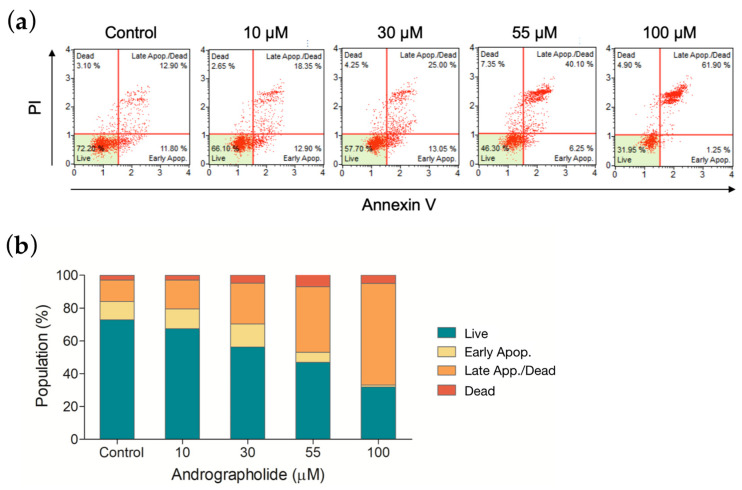
(**a**) Apoptosis was measured by flow cytometry following an annexin V/7-AAD binding assay. OEC-M1 cells were treated for 24 h using four different concentrations of andrographolide. (**b**) The mortality rate of OEC-M1 cells upon treatments of andrographolide for 24 h at the indicated concentrations.

The apoptotic effect was measured to distinguish the degree of cytotoxicity induced by andrographolide. OEC-M1 cells were treated with andrographolide at different concentrations, and the results were measured using an annexin V/7-AAD binding assay. Figure 6a shows a comparison of cells that are treated with andrographolide with the control group. As shown in the histogram of Figure 6b, andrographolide at 55 μM significantly induced OEC-M1 cells into the late stage of cell apoptosis and cell death. These results indicated that andrographolide significantly increases the number of apoptotic cells in a concentration-dependent manner.

## 4. Discussion

Patients with oral cancer frequently exhibit a local or regional invasion. This area is rich in vascular and lymphatic vessels. There is a greater propensity for oral cancer to metastasize to other sites in the body. Recurrent or metastatic oral cell carcinoma is usually treated with chemotherapy and surgical resection, but the mortality rate is high. Studies show that patients treated with a combination of cisplatin and 5-FU [37], which are standard chemotherapeutic drugs, demonstrate an increased overall response and disease progression decreases. While some patients demonstrate a specific improvement, other studies show clinical toxicity and limited efficacy. Oral cancers are invasive and highly proliferative, so an alternative therapeutic method is required. Drugs must follow an intrinsic apoptotic pathway, similarly to standard chemotherapeutic drugs.

Previous studies have shown that invasion and migration of other cancer cells are inhibited after treatment with andrographolide [38]. This study determines the cytotoxic effect of andrographolide on OCC-M1 cells. Cell viability assessed using alamarBlue indicates that 30 μM or higher concentration of andrographolide significantly displays an inhibitory effect. ECIS is a real-time label-free method used to monitor the changes in cell morphology and migration in response to the challenge of toxins [34,39,40]. The general impedance pattern for OEC-M1 cells consists of an initial stabilization phase, followed by another stabilization phase after the inoculation of cells (Figure 2b). The initial phase produces a low resistance of about 2 kΩ, and when cell confluency is achieved, resistance stabilizes to 5–6 kΩ. All OEC-M1 cells treated with andrographolide produce higher resistances than their initial value because the cells swell slightly after drug treatment. While the two higher concentrations, 55 and 100 μM, were distinguished from the control, the effects of the two lower concentrations, 10 and 30 μM, were negligible using the overall resistance measurement (Figure 2c).

The wound-healing assay results show that migratory rate is correlated with the inhibition effect of the drug. The results (Figure 4) show that the healing rate decreases significantly in a concentration-dependent manner. The highest concentration of andrographolide produces no recovery, as confirmed in parallel by light microscopy. Andrographolide has the optimal effect after the wounding assay. The lowest concentrations (10 μM) result in complete recovery at a stable resistance, similarly to confluency at 5 h post-wounding. These results demonstrate that andrographolide inhibits the migration of OEC-M1 cells.

The proliferation of cells decreases after drug treatment, and annexin V stain and flow cytometry results show that this depends on apoptosis. The results show a concentration-dependent increase in apoptotic cells for all four drugs compared to the control groups. The annexin V results show that it is expressed at higher levels at a 100 µM concentration of andrographolide, which agrees with ECIS results. These results agree with ECIS impedance results and are correlated with the cell viability assay for each concentration.

ECIS is a sensitive physiological and pharmacological test and is an ideal initial test platform for new drugs. This study shows that the most significant inhibitory effect is observed at a concentration of 100 µM of andrographolide, and cell apoptosis is induced. The morphological parameters at 55 µM obtained from ECIS (Figure 3b,c) are compared with annexin V data. The results show that the decrease in Rb and the increase in Cm are correlated with apoptosis-induced cytoplasmic shrinkage. This study proposes an impedance-based method for drug screening and cancer research.

## 5. Conclusions

In conclusion, this study investigated the cytotoxic effect of andrographolide on OEC-M1 cells. ECIS-related assays determine the drug’s effect on cell spreading, morphology, and migration. A 30 μM or higher concentration can shrink the cell’s chape and inhibit cell migration, leading to cell apoptosis. The results show that there is a concentration-dependent decrease in these measures in OEC-M1 cells that are treated with andrographolide. These results are also confirmed by biochemical assays of apoptosis-related markers using flow cytometry.

## Data Availability

The data underlying the results presented in this paper are not publicly available at this time but may be obtained from the authors upon reasonable request.

## Supplementary Materials

The following supporting information can be downloaded at: https://www.mdpi.com/article/10.3390/bios12050304/s1, Figure S1: A schematic of the ECIS setup. The lock-in amplifier supplies a 1-V AC signal through a 1 MΩ resistor, providing an approximately constant current of 1 µA across the sample. But the AC frequency can be freely selected for the impedance measurement. Only the small working electrode contributes significantly to the voltage measured by the amplifier across the sample; Figure S2.Time-series resistance of the cell-free electrode was measured at 4 kHz after addingculture medi-umas control(black) or 100 Mandrographolide (red)into the electrode well. Data obtainedfrom severalelec-trodewellswere averaged and represented as the mean ± standard error of the mean. (*n* = 4).

## References

[1] Blot W.J., McLaughlin J.K., Winn D.M., Austin D.F., Greenberg R.S., Preston-Martin S., Bernstein L., Schoenberg J.B., Stemhagen A., Fraumeni J.F. (1988). Smoking and drinking in relation to oral and pharyngeal cancer. Cancer Res..

[2] Guo S.-E., Huang T.-J., Huang J.-C., Lin M.-S., Hong R.-M., Chang C.-H., Chen M.-Y. (2013). Alcohol, betel-nut and cigarette consumption are negatively associated with health promoting behaviors in Taiwan: A cross-sectional study. BMC Public Health.

[3] Oliveira M.C., Menezes-Garcia Z., Henriques M.C., Soriani F.M., Pinho V., Faria A.M., Santiago A.F., Cara D.C., Souza D.G., Teixeira M.M. (2013). Acute and sustained inflammation and metabolic dysfunction induced by high refined carbohydrate-containing diet in mice. Obesity.

[4] Neville B.W., Day T.A. (2002). Oral cancer and precancerous lesions. CA A Cancer J. Clin..

[5] Huang S.H. (2013). Oral cancer: Current role of radiotherapy and chemotherapy. Med. Oral Patol. Oral Cir. Bucal.

[6] Metzstein M.M., Stanfield G.M., Horvitz H.R. (1998). Genetics of programmed cell death in C. elegans: Past, present and future. Trends Genet..

[7] Alberts K., Johnson A., Lewis J., Raff M., Roberts W.P., Walter P. (2008). Chapter 18 Apoptosis: Programmed cell death eliminates unwanted cells. Molecular Biology of the Cell.

[8] Majno G., Joris I. (1995). Apoptosis, oncosis, and necrosis. An overview of cell death. Am. J. Pathol..

[9] Griffiths G.J., Dubrez L., Morgan C.P., Jones N.A., Whitehouse J., Corfe B.M., Dive C., Hickman J.A. (1999). Cell damage-induced conformational changes of the pro-apoptotic protein Bak in vivo precede the onset of apoptosis. J. Cell Biol..

[10] Mikhailov V., Mikhailova M., Pulkrabek D.J., Dong Z., Venkatachalam M.A., Saikumar P. (2001). Bcl-2 prevents Bax oligomerization in the mitochondrial outer membrane. J. Biol. Chem..

[11] Kataoka T., Holler N., Micheau O., Martinon F., Tinel A., Hofmann K., Tschopp J. (2001). Bcl-rambo, a novel Bcl-2 homologue that induces apoptosis via its unique C-terminal extension. J. Biol. Chem..

[12] Marzo I., Brenner C., Zamzami N., Susin S.A., Beutner G., Brdiczka D., Rémy R., Xie Z.-H., Reed J.C., Kroemer G. (1998). The permeability transition pore complex: A target for apoptosis regulation by caspases and Bcl-2–related proteins. J. Exp. Med..

[13] Harris M., Thompson C. (2000). The role of the Bcl-2 family in the regulation of outer mitochondrial membrane permeability. Cell Death Differ..

[14] Zou H., Henzel W.J., Liu X., Lutschg A., Wang X. (1997). Apaf-1, a human protein homologous to C. elegans CED-4, participates in cytochrome c–dependent activation of caspase-3. Cell.

[15] Virág L., Marmer D.J., Szabó C. (1998). Crucial role of apopain in the peroxynitrite-induced apoptotic DNA fragmentation. Free Radic. Biol. Med..

[16] Kumar S., Harvey N.L. (1995). Role of multiple cellular proteases in the execution of programmed cell death. FEBS Lett..

[17] Halazonetis T.D., Gorgoulis V.G., Bartek J. (2008). An oncogene-induced DNA damage model for cancer development. Science.

[18] Israels E., Israels L. (2000). The cell cycle. Oncologist.

[19] Kirkin V., Joos S., Zörnig M. (2004). The role of Bcl-2 family members in tumorigenesis. Biochim. Biophys. Acta (BBA)-Mol. Cell Res..

[20] Szabo C. (2000). Cell Death: The Role of PARP.

[21] Tang W., Eisenbrand G. (1992). Andrographis paniculata (Burm. f.) Nees. Chinese Drugs of Plant Origin.

[22] Xia Y.-F., Ye B.-Q., Li Y.-D., Wang J.-G., He X.-J., Lin X., Yao X., Ma D., Slungaard A., Hebbel R.P. (2004). Andrographolide attenuates inflammation by inhibition of NF-κB activation through covalent modification of reduced cysteine 62 of p50. J. Immunol..

[23] Zhang Q.-Q., Ding Y., Lei Y., Qi C.-L., He X.-D., Lan T., Li J.-C., Gong P., Yang X., Geng J.-G. (2014). Andrographolide suppress tumor growth by inhibiting TLR4/NF-κB signaling activation in insulinoma. Int. J. Biol. Sci..

[24] Calabrese C., Berman S.H., Babish J.G., Ma X., Shinto L., Dorr M., Wells K., Wenner C.A., Standish L.J. (2000). A phase I trial of andrographolide in HIV positive patients and normal volunteers. Phytother. Res..

[25] Yu B.-C., Hung C.-R., Chen W.-C., Cheng J.-T. (2003). Antihyperglycemic effect of andrographolide in streptozotocin-induced diabetic rats. Planta Med..

[26] Rajagopal S., Kumar R.A., Deevi D.S., Satyanarayana C., Rajagopalan R. (2003). Andrographolide, a potential cancer therapeutic agent isolated from Andrographis paniculata. J. Exp. Ther. Oncol..

[27] Shen Y.-C., Chen C.-F., Chiou W.-F. (2000). Suppression of rat neutrophil reactive oxygen species production and adhesion by the diterpenoid lactone andrographolide. Planta Med..

[28] Bao Z., Guan S., Cheng C., Wu S., Wong S.H., Kemeny D.M., Leung B.P., Wong W.F. (2009). A novel antiinflammatory role for andrographolide in asthma via inhibition of the nuclear factor-κB pathway. Am. J. Respir. Crit. Care Med..

[29] Hidalgo M.A., Romero A., Figueroa J., Cortés P., Concha I.I., Hancke J.L., Burgos R.A. (2005). Andrographolide interferes with binding of nuclear factor-κB to DNA in HL-60-derived neutrophilic cells. Br. J. Pharmacol..

[30] Zhou J., Hu S.-E., Tan S.-H., Cao R., Chen Y., Xia D., Zhu X., Yang X.-F., Ong C.-N., Shen H.-M. (2012). Andrographolide sensitizes cisplatin-induced apoptosis via suppression of autophagosome-lysosome fusion in human cancer cells. Autophagy.

[31] Bothiraja C., Pawar A.P., Shende V.S., Joshi P.P. (2013). Acute and subacute toxicity study of andrographolide bioactive in rodents: Evidence for the medicinal use as an alternative medicine. Comp. Clin. Pathol..

[32] Tiruppathi C., Malik A.B., Del Vecchio P.J., Keese C.R., Giaever I. (1992). Electrical method for detection of endothelial cell shape change in real time: Assessment of endothelial barrier function. Proc. Natl. Acad. Sci. USA.

[33] Keese C.R., Giaever I. (1994). A biosensor that monitors cell morphology with electrical fields. Eng. Med. Biol. Mag. IEEE.

[34] Giaever I., Keese C.R. (1991). Micromotion of mammalian cells measured electrically. Proc. Natl. Acad. Sci. USA.

[35] Opp D., Wafula B., Lim J., Huang E., Lo J.-C., Lo C.-M. (2009). Use of electric cell–substrate impedance sensing to assess in vitro cytotoxicity. Biosens. Bioelectron..

[36] Lo C.-M., Keese C.R., Giaever I. (1993). Monitoring motion of confluent cells in tissue culture. Exp. Cell Res..

[37] Andreadis C., Vahtsevanos K., Sidiras T., Thomaidis I., Antoniadis K., Mouratidou D. (2003). 5-Fluorouracil and cisplatin in the treatment of advanced oral cancer. Oral Oncol..

[38] Lee K.-C., Chang H.-H., Chung Y.-H., Lee T.-Y. (2011). Andrographolide acts as an anti-inflammatory agent in LPS-stimulated RAW264. 7 macrophages by inhibiting STAT3-mediated suppression of the NF-κB pathway. J. Ethnopharmacol..

[39] Giaever I., Keese C.R. (1993). A morphological biosensor for mammalian cells. Nature.

[40] Liu Q., Yu J., Xiao L., Tang J.C.O., Zhang Y., Wang P., Yang M. (2009). Impedance studies of bio-behavior and chemosensitivity of cancer cells by micro-electrode arrays. Biosens. Bioelectron..

