# Supplementary material for: Real-Time Monitoring the Cytotoxic Effect of Andrographolide on Human Oral Epidermoid Carcinoma Cells
Source: Biosensors (Basel). 2022 May 6;12(5):304. doi: 10.3390/bios12050304 (PMC9138648; doi:10.3390/bios12050304)
Supplement: Supplementary file 1 [file biosensors-12-00304-s001.zip › biosensors-1673327-supplementary.pdf]

biosensors

MDPI

Article

Real-Time Monitoring the Cytotoxic Effect of Andrographolide on Human Oral Epidermoid Carcinoma Cells

Supplementary Materials

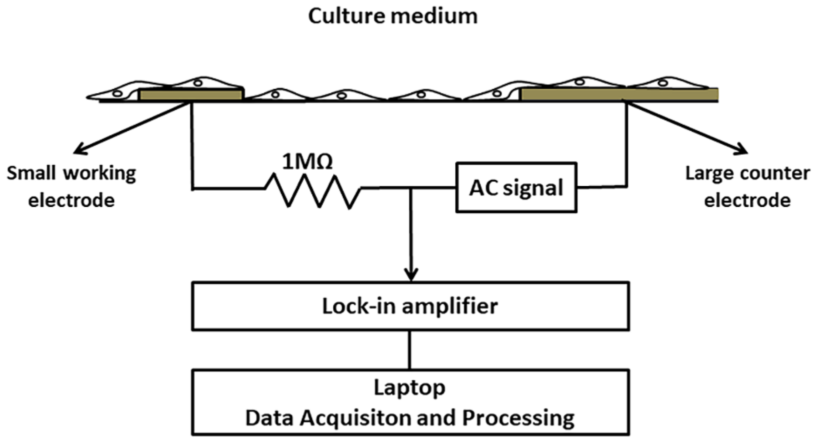

Figure S1. A schematic of the ECIS setup. The lock-in amplifier supplies a 1-V AC signal through a 1 MΩ resistor, providing an approximately constant current of 1 µA across the sample. But the AC frequency can be freely selected for the impedance measurement. Only the small working electrode contributes significantly to the voltage measured by the amplifier across the sample.

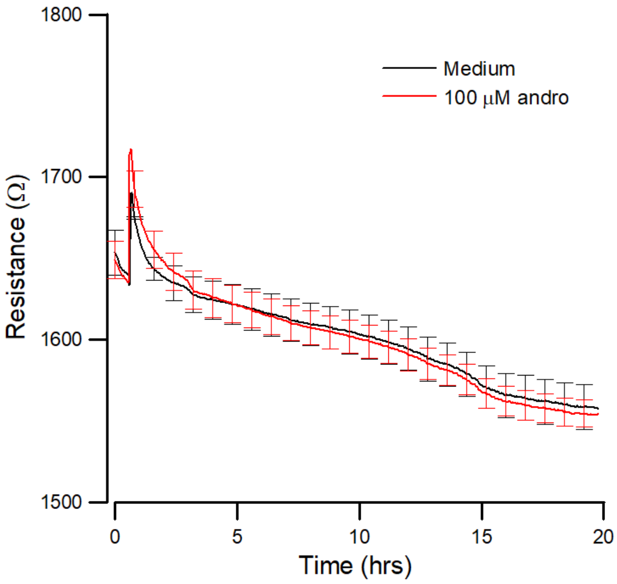

Figure S2. Time-series resistance of the cell-free electrode was measured at 4 kHz after adding culture medium as control (black) or 100 M andrographolide (red) into the electrode well. Data obtained from several electrode wells were averaged and represented as the mean ± standard error of the mean. (n = 4).
